# A New Species of the Genus *Achalinus* (Squamata: Xenodermidae) from the Dabie Mountains, Anhui, China

**DOI:** 10.3390/ani13040708

**Published:** 2023-02-17

**Authors:** Caiwen Zhang, Kai Liu, Ruyi Huang, Tingli Hu, Lei Yu, Ruolei Sun, Yucai Zhang, Jing Wen, Baowei Zhang

**Affiliations:** 1 School of Life Sciences, Anhui University, Hefei 230601, China; 2 Shanghai Universities Key Laboratory of Marine Animal Taxonomy and Evolution, Shanghai Ocean University, Shanghai 201306, China; 3 Administration of the Yaoluoping National Reserve, Anqing 246000, China; 4 Huoshan Forestry Bureau, Lu’an 237000, China

**Keywords:** *Achalinus dabieshanensis* **sp. nov.**, morphology, phylogenetics, taxonomy

## Abstract

**Simple Summary:**

A new species of odd-scaled snake in the genus *Achalinus* is described from Dabie Mountains Luan City, Anhui Province, China, based on one male and two female specimens. Bayesian inference and maximum likelihood analyses based on a mitochondrial DNA fragment (CO1) indicated the new taxon is different from its congeners (*p*–distance ≥ 9.4%). Morphologically, the new species can be diagnosed from the other species by a combination of 12 characters. The recognition of the new species brings the number of described *Achalinus* species to 22.

**Abstract:**

A new species of Xenodermid snake, *Achalinus dabieshanensis* **sp. nov.**, was described based on three specimens (two female and one male) collected from the Dabie Mountains of western Anhui Province. It can be distinguished from known congeners by a significant genetic divergence in the mitochondrial gene fragment COI (*p*-distance ≥ 9.4%) and the following combination of characteristics: (1) length of the suture between the internasals being distinctly shorter than between the prefrontals; (2) a single loreal; (3) dorsal scales strongly keeled, in 23 rows throughout the body; (4) two pairs of prefrontals; (5) six supralabials; (6) five infralabials; (7) temporals 2 + 2 + 3 (or 2 + 2 + 4); (8) 141–155 ventrals; (9) 45–55 subcaudals, unpaired; (10) anal entire; (11) weakly iridescent tinged, uniform, brown to black dorsum with vertebral scales and about three adjacent dorsal scales dark brown forming a longitudinal vertebral line from posterior margin of parietals to tail tip; (12) light brown venter, ventral shields wide, visible on both sides, light brown flanks, giving the appearance of a black subcaudal streak. The recognition of the new species increases the number of described *Achalinus* species to 22.

## 1. Introduction

The odd-scaled snakes (also known as burrowing snakes) of the genus *Achalinus* Peters, 1869 belong to the family Xenodermidae and currently include 21 species: *A. spinalis* (Peters, 1869), *A. rufescens* (Boulenger, 1888), *A. formosanus* (Boulenger, 1908), *A. werneri* (Van Denburgh, 1912)*, A. niger* (Maki, 1931), *A. ater* (Bourret, 1937), *A. meiguensis* (Hu and Zhao, 1966), *A. hainanus* (Huang, 1975), *A. jinggangensis* (Zong and Ma, 1983), *A. juliani* (Ziegler et al., 2019), *A. emilyae* (Ziegler et al., 2019), *A. timi A. timi* (Ziegler et al., 2019), *A. yunkaiensis* (Wang et al., 2019), *A. pingbianensis* (Li et al., 2020), *A. tranganensis* (Luu et al., 2020), *A. zugorum* (Miller et al., 2020), *A. panzhihuaensis* (Hou et al., 2021), *A. yangdatongi* (Hou et al., 2021), *A. dehuaensis* (Hou et al., 2021) and *A. ningshanensis* (Yang et al. 2022) [1,2,3,4,5,6,7,8,9,10,11,12,13,14,15,16,17]. Morphologically, all species of the genus *Achalinus* have conservative morphological characters, including a slender, cylindrical body, a head that is slightly distinct from the neck, small eyes, and an entire anal [18,19]. Although most of their morphological characteristics overlap between species [20], some significant differences in color patterns and squam characteristics have been recognized. The presence of a longitudinal vertebral line from the posterior margin of the parietal to the tail tip, the numbers of infralabials and supralabials, and the lengths of the sutures between the internasals and the prefrontals differ. Recent phylogenetic studies have also supported the species divisions within the genus *Achalinus* [21,22]. The genus *Achalinus* is widely distributed in eastern and southeastern Asia, ranging from northern Vietnam to southwestern China, and partly into Japan. Presently, 16 species occur in China, of which ten are endemic to the country [14,15,17].

During recent herpetological surveys of the Dabie Mountains, we collected three odd-scaled snake specimens that differed from all other species of known snakes in their morphology and color pattern characteristics. According to the diagnostic characteristics of the genus *Achalinus*, as indicated by Smith and Zhao et al. [18,19], the specimens could be assigned to this genus. Further morphological examinations and molecular analyses showed that these specimens represent a separate evolutionary lineage that can be distinguished from all recognized species. Herein, we describe these specimens as a new species of the genus *Achalinus*.

## 2. Materials and Methods

### 2.1. Sampling

We obtained three odd-scaled snake samples from the Yaoluoping Nature Reserve and Fuziling Provincial Reserve in the Dabie Mountains, Anhui Province, China. The specimens were collected in the field and fixed in 75% ethanol. Liver tissue samples were separately transferred to 95% ethanol. The specimens have been deposited in the Biological Museum of Anhui University, Anhui, China. Permission for the field surveys in the Dabie Mountains was granted by the Huoshan Forestry Bureau (No. HFB20180801) and Management Office of the Yaoluoping National Reserve (No. YNR 20190503).

### 2.2. DNA Sequencing

Tissue samples from three individuals of the new specimens were used for the phylogenetic analyses. DNA was extracted from the liver samples using phenol/chloroform extraction [23]. The partial mitochondrial gene fragment of the cytochrome c oxidase 1 gene (COI) was amplified using the primer pair RepCOIF (5′-TNT TMT CAA I ACC ACA AAG A-3′) and RepCOIR (5′-ACT TCT GGR TGK CCA AAR AAT CA-3′) [24]. Experimental primer pairs were synthesized and provided by General Biosystems (Anhui) Co., Ltd. (Chuzhou, China). The PCR conditions followed those described by Nagy et al. (2012) [24]. The PCR products were sequenced by General Biosystems (Anhui) Co., Ltd. The sequences were edited and assembled using SeqMan (DNASTAR, Lasergene v7.1) and then aligned using Clustal X 1.8 [25] in MEGA6 [26]. Sequences with insertions or deletions (indels) were sequenced at least twice with both the forward and reverse primers to confirm the variations. All sequences were deposited in GenBank (Table 1).

### 2.3. Phylogenetic Analyses

To explore the phylogenetic position of the samples in the genus *Achalinus*, COI sequences from 21 snakes, including three outgroups, *Fimbrios klossi*, *Parafimbrios lao,* and *P. vietnamensis*, were retrieved from GenBank for our analyses (Accession number listed in Table 1) [16,17]. The nucleotide substitution models were tested in MrModeltest 2.3 using Akaike information criteria [27], and the GTR + G + I model was found to be the best fit. Bayesian analyses were conducted using MrBayes 3.2.4 [28]. Three independent runs with four Markov Chain Monte Carlo were performed based on the models selected, starting from a random tree; each run consisted of a total of 3 × 10^6^ generations, sampled every 2000 generations. The first 25% of the samples were discarded as burn-in. The remaining trees were used to construct a 50% majority rule consensus tree. The results were analyzed in Tracer v1.7 to assess convergence and ensure effective sample sizes (≥200) for all parameters [29]. Maximum likelihood analyses were executed using IQ-TREE 2 (Minh et al., 2020), and the support of the tree was by bootstrap resampling with 1000 nonparametric bootstrap replicates. Phylogenetic trees were visualized and edited using FigTree v1.4.2 [30]. We also calculated the uncorrected pairwise genetic distances (*p*-distance) using MEGA 6 [25].

### 2.4. Morphometric Measurements

Morphometric measurements to the nearest 0.1 mm were taken using dial calipers, following Zhao et al. (1998) [19]. The abbreviations of morphological characteristics used in the text are as follows: total length (TL, from snout tip to tail end); snout-vent length (SVL, from cusp of snout to anterior margin of cloacae opening); tail length (TaL, from posterior margin of cloacae opening to tip of tail); head length (HL, from snout tip to posterior margin of mandible); head width (HW, at the widest part of the head); head height (HH, at the highest part of the head); length of loreal (LeL, at the longest part of the loreal); height of loreal (HiL, at the highest part of the loreal); length of anterior section of nasal (LaSN); length of posterior section of nasal (LpSN); length of suture between prefrontals (LSBP); length of suture between internasals (LSBI); preoculars (PrO); postoculars (PtO); supraoculars (SpO); supralabials (SPL); infralabials (IFL); anterior temporals (aTMP); middle temporals (mTMP); posterior temporals (pTMP); ventral scales (V); and subcaudals (SC). Dorsal scale rows (DSR) were counted at two head lengths behind the head, mid-body, and two head lengths before the vent. Bilateral scale counts are given as left/right. Sex identification was performed by inspecting the presence or absence of hemipenes. In this study, while comparing the undescribed species with *A. rufescens*, the most widespread species, we used its latest description of *A. rufescens* rather than descriptions in the early literature, because it was very likely that multiple cryptic species will be involved in them [10]. In the morphometric analysis, we focused on the species that clustered most closely with the new specimen in the phylogenetic trees. Morphological data for the *Achalinus* species were obtained from the relevant literature sources [1,2,3,4,5,6,7,8,9,10,11,12,13,14,15,16,17].

The electronic version of this articIe in Portable Document Format (PDF) will represent a published work according to the International Commission on Zoological Nomenclature (ICZN), and hence the new names contained in the electronic version are effectively published under that Code from the electronic edition alone. This published work and the nomenclatural acts it contains have been registered in ZooBank, the online registration system for the ICZN. The ZooBank LSIDs (Life Science Identifiers) can be resolved, and the associated information can be viewed through any standard web browser by appending the LSID to the prefix http://zoobank.org/ (accessed on 22 October 2022). The LSID for this publication is urn:lsid:zoobank.org:pub: 1E1842EA-CB20-46FA-AA0D-3D35017814D4.

## 3. Results

### 3.1. Phylogenetic Relationship

**Figure 1:**
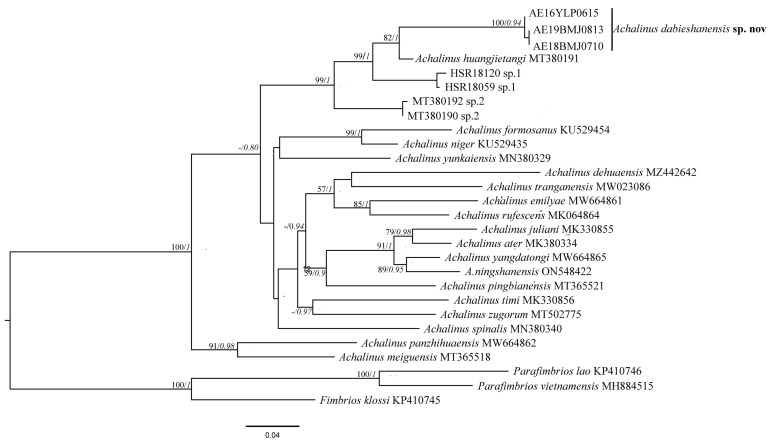
Maximum likelihood (ML) tree of the genus *Achalinus* reconstructed based on DNA sequences of segments of the COI genes. ML bootstrap support/Bayesian posterior probability is denoted beside each node, and the symbol “-” indicates values below 50%. Sample information is provided in Table 1.

The alignment contained 657 nucleotide base pairs (bp) sequences. The Bayesian inference (BI) and Maximum likelihood (ML) phylogenetic results (Figure 1) were largely concordant concerning the relationships among species within the major clades of *Achalinus*. The *Achalinus* lineages formed a monophyletic group with high support values (BI, PP = 1.00; ML, BS = 100%). These three specimens from the Dabie mountain were nested within the *Achalinus* genus and the independence of our collections and their affinity to *A. huangjietangi* were also strongly supported (BI, PP = 1.00; ML, BS = 82%). In addition, these new samples have distinct branch lengths compared with the adjacent clade of *A. huangjietangi* (HSR18030 collection number).

The uncorrected *p*-distance among all species within the genus *Achalinus* ranged from 6.1% to 21.7% (Table 2). The maximum genetic distance was found between the new species and *A. formosanus* (21.7%), and the minimum one was found between the new species and *A. huangjietangi* (9.4%) (Table 2). The genetic distance between the new samples and *A. huangjietangi* (9.4%) is greater than the lowest three ones: (7.5% between *A. ater* and *A. juliani*, 6.5% between *A. yangdatongi* and *A. juliani*, and 6.1% between *A. ningshanensis* and *A. yangdatongi*).

Given that these specimens possess obvious monophyletic structures in the molecular phylogenetic tree, adding the significant molecular distance, unique geographical distribution, and prominent morphological differences from congenetic species, they are described as a new species below.

### 3.2. Taxonomic Account

**Figure 2:**
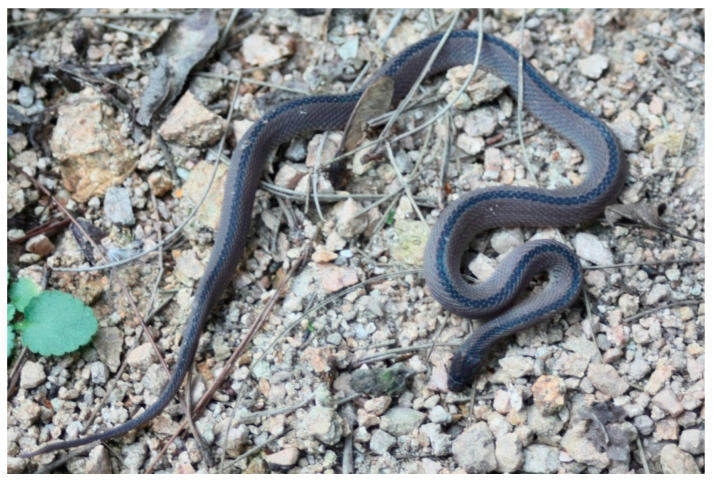
General view of the holotype of *Achalinus dabieshanensis* **sp. nov.** in life (Photography by Kai Zhao).

**Figure 3:**
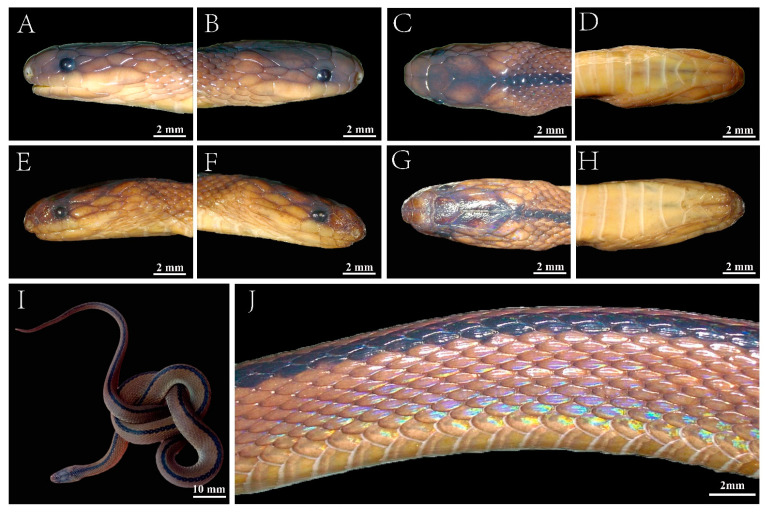
The general aspect of the adult female holotype (AHU 2018-EE-0710) and the adult male paratype (AHU 2016-EE-0615) of *Achalinus dabieshanensis* **sp. nov**. (**A**–**D**) the holotype left and right and dorsal and ventral view of head (showing length of the suture between internasals shorter than it between prefrontals, a single loreal, six supralabials, five infralabials and temporals 2 + 2 + 3); (**E**–**H**) the paratype left and right and dorsal and ventral view of head; (**I**) Overall view of the preserved holotype; (**J**) the holotype dorsal view of midbody (showing dorsum iridescent brown with three scales wide dorsal black line). Photos by Lihua Huang and Caiwen Zhang.

*Achalinus dabieshanensis* **sp. nov.** Zhang, Liu, Huang and Zhang (Figure 2 and Figure 3A–F; and Table 3).

**Holotype:** AHU 2018-EE-0710 (Figure 2 and Figure 3A–F), an adult female, collected by Tingli Hu, Zhen Xu, Ruolei Sun, and Guotao Chen on 10 July 2018 from Fuziling Provincial Reserve in the Dabie Mountains (30°57′47.88″ N, 116°04′37.20″ E; 1361 m a.s.l.), Huoshan County, Luan City, Anhui Province, China.

**Paratypes:** AHU 2016-EE-0615 (Figure 3A–D), an adult male, collected by Lei Yu and Tao Pan on 15 June 2016 from the Yaoluoping Nature Reserve in the Dabie Mountains (30°59′05.99″ N, 116°04′45.20″ E; 1051 m a.s.l.), Yuexi County, Anqing City, Anhui Province, China. AHU 2019-EE-0813, a female collected by Caiwen Zhang and Haohao Ma on13 August 2019 from the same locality as the holotype (31°11′6.72″ N, 116°04′38.28″ E; 635 m a.s.l).

### 3.3. Diagnosis

A new species of *Achalinus* with: (1) weakly iridescent tinged, uniform brown dorsum with vertebral scales and about three adjacent dorsal scales dark brown, forming longitudinal vertebral line from posterior margin of parietals to tail tip; (2) light brown venter; (3) relatively short tail, TaL/TL ratio 16.8–22.3%; (4) suture length between internasals distinctly shorter than between prefrontals; (5) one loreal (height/length ratio 0.79–0.89); (6) six supralabials, 4th and 5th widely in contact with eye; (7) temporals 2 + 2 + 3 (or 2 + 2 + 4), two elongated anterior temporals in contact with eye; (8) five infralabials, two pairs of chin shields, first three infralabials touching first pair of chin shields; (9) 141–155 ventrals, 45–55 subcaudals, not paired; (10) dorsal scales in 23 rows throughout, strongly keeled, but outermost rows on both sides smooth and significantly enlarged; (11) anal entire.

### 3.4. Comparisons

**Figure 4:**
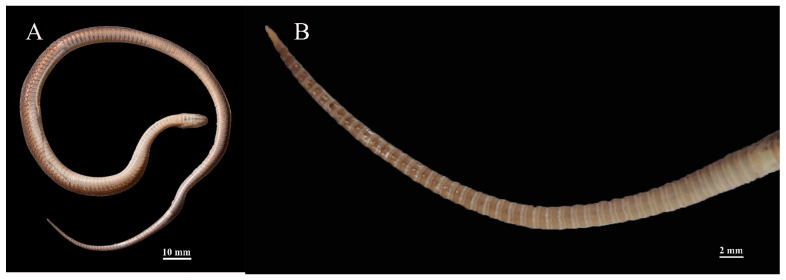
Ventral of the adult female holotype (AHU 2018-EE-0710). (**A**) ventral; (**B**) close-up of caudal ventral (showing absence of a dark streak in the middle of caudal ventral).

*Achalinus dabieshanensis* **sp. nov.** differs from all other species of *Achalinus*, except *A. formosanus*, *A. huangjietangi*, *A. niger*, and *A. spinalis* by having internasal suture lengths distinctly shorter than the lengths of the suture between the prefrontals (vs. other species of *Achalinus*, length of suture between the internasals distinctly longer than or equal to that between the prefrontals). In addition, it differs from all other species of *Achalinus*, except *A. dehuaensis*, *A. emilyae*, *A. hainanus*, *A. huangjietangi*, *A. rufescens*, *A. meiguensis,* and *A. ningshanensis*, by having five infralabials (other species of *Achalinus* have six or seven). Thus, these characteristics are not repeated in the detailed comparisons that follow. At first glance, the color pattern and dorsal scale-row formula of *A. dabieshanensis* **sp. nov.** are most similar to those of *A. huangjietangi*, *A. rufescens,* and *A. yunkaiensis*. However, *Achalinus dabieshanensis* **sp. nov.** can be easily distinguished from *A. huangjietangi* by having fewer ventral scales (141–155 vs. 157–170 in *A. huangjietangi*) and the absence of a dark streak in the middle of caudal ventral (Figure 4). The new species can be distinguished from *A. rufescens* by two anterior pairs of chin shields (vs. three pairs of chin shields in *A. rufescens*) and two anterior temporals in contact with the eye (vs. only the upper anterior temporal in contact with the eye in *A. rufescens*), from *A. yunkaiensis* by having fewer ventral scales (45–55 vs. 56–59 in *A. yunkaiensis*) and the obvious three-scale-wide mid-dorsal dark longitudinal line.

*Achalinus dabieshanensis* **sp. nov.** can be differentiated from *A. ater* by having fewer ventral scales (141–155 vs. 160–170); from *A. dehuaensis* by having two anterior temporals (vs. only one), fewer subcaudals (45–55 vs. 63–81); from *A. emilyae* by having fewer subcaudals (45–55 vs. 60–65) and fewer ventral scales (141–155 vs. 157–166); from *A. formosanus* by having fewer DSR (23//23//23 vs. 27–29//25–27//25), fewer subcaudals (45–55 vs. 61–83), and fewer ventral scales (141–155 vs. 158–169); from *A. hainanus* by having two anterior temporals (vs. only one), fewer subcaudals (45–55 vs. 67–69), fewer ventral scales (141–155 vs. 165–168), and a relatively short tail (TaL/TL 16.8–22.3% vs. 26–27%); from *A. jinggangensis* by having the loreal not being fused with the prefrontal (vs. loreal fused with the prefrontal) and fewer ventral scales (141–155 vs. 156–164); from *A. juliani* by having fewer subcaudals (45–55 vs. 77–91) and fewer ventral scales (141–155 vs. 163–179); from *A. meiguensis* by having internasals (vs. lacking internasals), lacking postocular (vs. having postocular), and more DSR both anteriorly and posteriorly (23 vs. 21 or 23 and 23 vs. 19, respectively); from *A. niger* by having fewer ventral scales (141–155 vs. 169–185), fewer DSR anteriorly (23 vs. 25), and fewer middle temporals (23 vs. 25); from *A. ningshanensis* by having a relatively long tail (TaL/TL 16.8–22.3% vs. 12–16%) and fewer ventral scales (141–155 vs. 171); from *A. pangzhihuaensi* by having internasals (vs. lacking internasals), lacking postocular (vs. having postocular), and more DSR posteriorly (23 vs. 19), fewer ventral scales (141–155 vs. 160), a relatively short tail (TaL/TL 16.8–22.3% vs. 24.6%), and fewer subcaudals (45–55 vs. 73); from *A. pingbianensis* by having fewer supralabials (six vs. seven), fewer subcaudals (45–55 vs. 56), and fewer ventral scales (141–155 vs. 160); from *A. spinalis* by having two anterior temporals in contact with eye (vs. upper anterior anterior broadly in contact with eye) and fewer infralabials (five vs. six); from *A. tranganensis* by having fewer DSR anteriorly (23 vs. 25), a relatively short tail (TaL/TL 16.8–22.3% vs. 25%), fewer subcaudals (45–55 vs. 73+), and fewer ventral scales (141–155 vs. 171); from *A. werner* by having a relatively short tail (TaL/TL 16.8–22.3% vs. 25%), fewer subcaudals (45–55 vs. 67–98), and fewer ventral scales (141–155 vs. 157–191); from *A. yangdatongi* by having more DSR posteriorly (23 vs. 19), fewer vetral scales (141–155 vs. 161), a relatively short tail (TaL/TL 16.8–22.3% vs. 26.2%), and fewer subcaudals (45–55 vs. 82); and from *A. zugorum* by having fewer infralabials (five vs. seven), fewer DSR anteriorly (23 vs. 25), fewer subcaudals (45–55 vs. 70), and fewer ventral scales (141–155 vs. 173). Comparisons between the new species and its congeners are summarized in Table 4.

### 3.5. Description of Holotype

Adult female with total length 316 mm (SVL 263 mm, TaL 53 mm); tail relatively short, TaL/TL ratio 16.8%; body slender, cylindrical; HL 9.5 mm, HW 7.2 mm, HH 3.5 mm; head indistinct from neck; eye small with the vertically elliptical pupil; rostral small, triangular, scarcely visible from above; HL/RW ratio 0.7; internasal suture (0.7 mm) about half length of prefrontal suture (1.2 mm).

Nostril in anterior part of nasal, posterior margin of nostril with distinct nostril cleft, posterior section of nasal vertically rectangular, posterior section nearly half as long as anterior section, LaSN/LpSN 0.4; single pentagonal frontal, nearly straight anteriorly, slightly broader than long, pointed backwards, much shorter than parietals; single loreal, HiL/LeL ratio 0.8, extending from nasal to eye; single supraocular, elongated, twice as wide as high; two anterior temporals, elongated, upper one smaller, widely in contact with eye, lower one narrowly in contact with eye; two elongated middle temporals, upper one much larger, lower one in contact with 6th supralabial, not in contact with elongated anterior temporals on left, only tip in contact with anterior temporals on right; three elongated posterior temporals, uppermost one significantly enlarged (super-temporal), surrounding the parietal; each parietal bordered by elongated nuchal; nuchals separated from each other behind super-temporals by one small intertemporal nuchal scale; 2nd nuchal about twice size of 1st; six supralabials, 1st smallest, 4th and 5th widely in contact with eye, 6th longest and largest; 3rd and 4th in broad contact with loreal; one mental, followed by five infralabials with first pair in contact with each other; two pairs anterior and posterior chin shields in contact with 3rd infralabial; posterior pair of chin shields smaller, length of suture between 1st pair twice that between 2nd pair; dorsal scales lanceolate and feebly keeled; dorsal scales in 23 rows throughout body, outermost rows on both sides smooth and significantly enlarged; 155 ventrals, distinctly rounded laterally; 45 subcaudals, not paired; anal entire.

The coloration of the holotype in life Dorsal surface is uniform iridescent brown, with a longitudinal dark brown vertebral line, a width of about 3 DSR, from posterior margin of parietals to tail tip, ventrals light brown; margins of all scales grayish white; coloration of supralabials and temporal regions much lighter; iris dark brown, pupil black.

### 3.6. Intraspecific Morphological Variations

The measurements, scale counts, body proportions, and squamae details are listed in Table 3. All paratypes are morphologically very similar to the holotype except that: (1) The adult male (AHU 2016-EE-0615) possessed a significantly larger body size, TL 376.2 mm, TaL 84 mm, TaL/TL radio 22.3% (vs. TL 316 mm, TaL 53 mm, TaL/TL radio 16.8 % in female holotype; TL 226 mm, TaL 47 mm, TaL/TL radio 17.7% in female paratype); (2) the adult male (AHU 2016-EE-0615) and second female (AHU 2019-EE-0813) had TMP 2 + 2 + 4/2 + 2 + 4 (vs. TMP 2 + 2 + 3/2 + 2 + 3 in holotype); and (3) the LSBI/LSBP radio was 0.8 in AHU 2019-EE-0813 (vs. an LSBI/LSBP ratio of 0.7 in the holotype and paratype AHU 2019-EE-0615).

### 3.7. Etymology

The specific epithet, *Achalinus dabieshanensis* **sp. nov.**, refers to the distribution of the new species in the Dabie Mountains in Anhui, China. We suggest the English name would be the “Dabie Mountains Odd-scaled Snake” or “Dabie Mountain Burrowing Snake” and the Chinese name “大别山脊蛇 (Dà Bié Shān Jǐ Shé)”.

### 3.8. Distribution and Habitat

**Figure 5:**
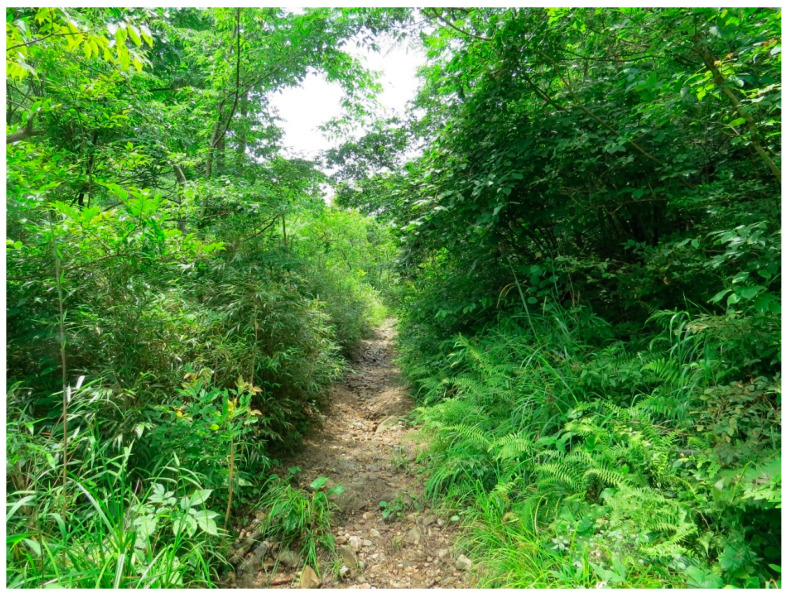
Habitat of *Achalinus dabieshanensis* **sp. nov**. in its type locality in the Dabie Mountains, Anhui, China (Photography by Baowei Zhang).

Currently, *Achalinus dabieshanensis* **sp. nov.** is only known from its type-locality, Fuziling Provision Reserve, Yaoluoping Nature Reserve, and an adjacent area in the Dabie Mountains, Anhui, China (Figure 5). The new species was discovered in the leaf litter of a well-preserved montane evergreen deciduous broad-leaved mixed forest (635–1361 m a.s.l.).

## 4. Discussion

*Achalinus* is an ancient group, which diverged from its closest related genus approximately 77.4 million years ago, and is widely distributed in China, Vietnam, and Japan now [4,18,19,31]. However, the morphological characteristics of the genus *Achalinus* are relatively conservative, such as its color, number of scales, etc. [20], which require detailed morphological differences comparison and more molecular evidence to distinguish species [14,20]. Thus, for *Achalinus*, slight morphological differences often play key roles in species delimitation. For example, in 2019, *A. yunkaiensis* was discovered in Guangdong; it only differs from *A. spinalis* by comparing the length of suture between the internasals and that between the prefrontals [10]. Another example is that *A. huangjietangi* only differs from *A. yunkaiensis* by a dark streak in the middle of the caudal ventral [14]. In addition, *A. huangjietangi* differs from *A. spinalis* by the number of anterior temporals in contact with the eye and a dark streak in the middle of the caudal ventral [14]. In contrast, there are often very substantial genetic differentiations between these species. Given the significant molecular distance within the specimens of *A. huangjietangi*, we suspect that there are one or more cryptic species to be further investigated; because of the lack of the morphological data, we could not further investigate the taxonomic relationship within those specimens [14]. Temporarily in this study, they are labeled as sp. 1 and sp. 2 (Figure 1).

In this study, ML and BI trees showed that *A. dabieshanensis* **sp. nov.** is the sister to *A. huangjietangi* (Figure 1), and these two lineages have distinct morphologies (Figure 3, Table 4). Moreover, the difference between *A. dabieshanensis* and *A. huangjietangi* is also reflected in their significant genetic divergences (*p*-distance = 9.4%, Table 2); their genetic distance is higher than that among many species in the genus. In general, *Achalinus* species tend to inhabit wetter, more mountainous areas with low dispersal ability [9], so large rivers may form insurmountable geographical barriers [13]. In this study, the Yangtze River acted as the geographical barrier separating *A. huangjietangi* from the *A. dabieshanensis* **sp. nov.**. Similar situations have been reported in adjacent species, such as *A. timi* and *A. zugorum*; they were separated by the Red River [13]. Therefore, the limited dispersal ability and geographical barriers may have probably led to species divergence between *A. huangjietangi* and *A. dabieshanensis* **sp. nov.** Based on the molecular and morphological evidence, we think that *A. dabieshanensis* **sp. nov.** should be considered a valid species.

The discovery of *A. dabieshanensis* **sp. nov.** extends the distribution of the genus northward to Dabie Mountain in the lower reaches of the Yangtze River. To date, many new vertebrate species have been discovered in the Dabie Mountains [32,33,34,35,36,37]; the discovery of the *A. dabieshanensis* **sp. nov.** further suggests that species diversity in the Dabie Mountains remains underestimated to some extent. At present, based on the collection sites of the three new specimens, it is speculated that *A. dabieshanensis* **sp. nov.** may be distributed throughout the Dabie Mountains. Given that the secretive nature of odd-scaled snakes makes their discovery largely serendipitous [10], we cannot make exact judgments about the abundance and population status of the new species in the Dabie Mountain area. Therefore, it is difficult to assess the risk of extinction of this species for the time being. We recommend classifying *A. dabieshanensis* **sp. nov.** as Data Deficient (DD) on the IUCN Red List. It is worth noting that human activities in the Dabie Mountains in recent years, including vegetation destruction, road construction, and artificial surface expansion [38], may threaten this species.

## 5. Conclusions

A new species of *Achalinus*, *Achalinus dabieshanensis* **sp. nov.**, is described based on three specimens collected from the Dabie Mountains of western Anhui Province. It appears to be widespread in the Dabie Mountains. The discovery of new species made the members of the genus *Achalinus* distribution area extend northward to the Dabie Mountain in the lower reaches of the Yangtze River. However, their discovery is largely accidental, which makes it difficult for us to make accurate judgments on the abundance and population status of this new species in the Dabie Mountains. Further investigations will be necessary to assess the risk of extinction of this species.

## Figures and Tables

**Table 1:** Samples used for molecular phylogenetic analysis in this study.

ID	Species	Specimen Voucher	Locality	GenBank Number
1	*Achalinus dabieshanensis* **sp. nov.**	AHU2016EE0615	Yaoluoping Nature Reserve, Anhui, China	MW316597
2	*Achalinus dabieshanensis* **sp. nov.**	AHU2018EE0710	Fuziling Provincial Reserve, Anhui, China	MW316598
3	*Achalinus dabieshanensis* **sp. nov.**	AHU2019EE0813	Yaoluoping Nature Reserve, Anhui, China	MW316596
4	*Achalinus ater*	SYS r000852	Anjiangping, Guangxi, China	MK064760
5	*Achalinus dehuaensis*	YBU 13013	Dehua, Fujian, China	MZ442642
6	*Achalinus emilyae *	IEBR 4465	Hoanh Bo District, Quang Ninh, Vietnam	MK330857
7	*Achalinus formosanus*	RN 2004	Taiwan Province, China	KU529454
8	*Achalinus huangjietangi*	HSR18030	Huangjialing Village, Anhui, China	MT380191
9	*Achalinus juliani*	IEBR A.2018.9	Ha Lang District, Cao Bang, Vietnam	MK330855
10	*Achalinus meiguensis*	GP836	Mianyang, Sichuan, China	MT365518
11	*Achalinus niger*	RN 1159	Taiwan Province, China	KU529435
12	*Achalinus ningshanensis*	HSR19232	Ningshan County, Shaanxi Province, China	ON548423
13	*Achalinus panzhihuaensis*	KIZ 040189	Hongbao, Yanbian, Sichuan, China	MW664862
14	*Achalinus pingbianensis*	YBU 18273	Honghe, Yunnan, China	MT365521
15	*Achalinus rufescens*	SYS r001527	Heishiding, Guangdong, China	MK064864
16	*Achalinus spinalis*	SYS r001327	Mt. Badagong, Hunan, China	MN380340
17	*Achalinus timi*	IEBR A.2018.10	Thuan Chau District, Son La, Vietnam	MK330856
18	*Achalinus tranganensis*	VNUF R.2018.21	Ninh Binh Province, Vietnam	MW023086
19	*Achalinus yangdatongi*	KIZ 034327	Wenshan Nature Reserve, Yunnan, China	MW664865
20	*Achalinus yunkaiensis*	SYS r001443	Guangdong, China	MN380329
21	*Achalinus zugorum*	IEBR 4698	Ha Giang Province, Vietnam	MT502775
	**Out group**			
22	*Fimbrios klossi*	IEBR A.2013.56	Gia La, Vietnam	KP410745
23	*Parafimbrios lao*	MNHN 2013.1002	Louangphabang, Laos	KP410746
24	*Parafimbrios vietnamensis*	IEBR A.2018.7	Lai Chau, Vietnam	MH884515

**Table 2:** Uncorrected *p*-distances among the *Achalinus* species based on partial mitochondria COI gene.

ID	Species	1	2	3	4	5	6	7	8	9	10	11	12	13	14	15	16	17	18	19
1	*A. dabieshanensis* **sp. nov.**																			
2	*A. huangjietangi*	**0.094**																		
3	*A. juliani*	0.185	0.169																	
4	*A. ater*	0.174	0.171	**0.068**																
5	*A. yangdatongi*	0.191	0.165	**0.075**	**0.065**															
6	*A. rufescens*	0.185	0.172	0.129	0.140	0.127														
7	*A. emilyae*	0.209	0.168	0.159	0.132	0.147	0.112													
8	*A. spinalis*	0.186	0.150	0.161	0.172	0.160	0.141	0.173												
9	*A. yunkaiensis*	0.168	0.138	0.149	0.142	0.132	0.154	0.147	0.134											
10	*A. formosanus*	**0.217**	0.179	0.144	0.161	0.163	0.163	0.158	0.160	0.139										
11	*A. niger*	0.196	0.156	0.130	0.131	0.147	0.145	0.149	0.143	0.134	0.080									
12	*A. pingbianensis*	0.173	0.144	0.133	0.127	0.124	0.143	0.154	0.148	0.125	0.164	0.139								
13	*A. zugorum*	0.171	0.162	0.154	0.154	0.136	0.160	0.149	0.152	0.123	0.151	0.151	0.117							
14	*A. timi*	0.186	0.168	0.167	0.149	0.146	0.168	0.142	0.161	0.157	0.151	0.134	0.131	0.152						
15	*A. tranganensis*	0.175	0.149	0.156	0.146	0.143	0.139	0.143	0.170	0.153	0.196	0.164	0.148	0.134	0.160					
16	*A. meiguensis*	0.196	0.173	0.187	0.167	0.194	0.208	0.175	0.175	0.170	0.173	0.149	0.191	0.162	0.174	0.174				
17	*A. panzhihuaensis*	0.191	0.173	0.183	0.187	0.178	0.180	0.197	0.182	0.178	0.186	0.173	0.170	0.174	0.177	0.187	0.123			
18	*A. ningshanensis*	0.211	0.199	0.096	0.080	**0.061**	0.137	0.159	0.176	0.154	0.168	0.147	0.128	0.144	0.151	0.173	0.195	0.204		
19	*A. dehuaensis*	0.211	0.189	0.165	0.191	0.158	0.144	0.178	0.162	0.168	0.187	0.175	0.168	0.164	0.176	0.152	0.201	0.174	0.189	

**Table 3:** Measurements, scale counts, body proportions, and squamae characters of the sample of *Achalinus dabieshanensis* **sp. nov**.

Voucher	AHU 2016-EE-0615	AHU 2018-EE-0710	AHU 2019-EE-0813
Sex	male	female	Male
TL	376	316	266
SVL	287	263	217
TaL	84	53	47
TaL/TL	22.3%	16.8%	17.7%
HW	5.6	5.3	4.8
HL	11.2	9.5	8.7
HH	4.5	3.5	3.1
HW/HL	0.5	0.6	0.6
SPL	6	6	6
SPL–Lorea	3rd–4th	3rd–4th	3rd–4th
IFL	5	5	5
Loreal	1	1	1
HiL	1.1	1.0	0.9
LeL	1.5	1.2	1.1
HiL/LeL	0.7	0.8	0.8
LSBP	1.2	0.8	0.9
LSBI	1.8	1.2	1.1
LSBP/LSBI	0.7	0.7	0.8
LaSN	0.3	0.3	0.3
LpSN	0.8	0.7	0.5
LaSN/LpSN	0.4	0.4	0.6
SpO	1	1	1
TMP	2 + 2 + 4/2 + 2 + 4	2 + 2 + 3/2 + 2 + 3	2 + 2 + 4/2 + 2 + 4
Elogate aTMP	2nd	2nd	2nd
Elogate mTMP	1st	1st	1st
Elogate pTMP	1st	1st	1st
DSR	23 + 23 + 23	23 + 23 + 23	23 + 23 + 23
V	141	155	151
SC	55	45	46

**Table 4:** Comparisons of main morphological characters among *Achalinu dabieshanensis* **sp. nov.** species.

Species	TL Max.	SVL Max.	TaL Max.	TaL/TL	Int. Fus.	SPL	TMP	Loreal	IFL	aTMP [Eye Contact]	LSBI vs. LSBP	Ant DSR	Mid DSR	Post DSR	V	Post DSR
*Achalinus dabieshanensis* **sp. nov**	376	287	84	16.8–22.3%	no	6	2 + 2 + 3 (4)	1	5	2	<	23	23	23	141–155	45–55
*A. ater*	401	-	70	19–22%	no	6	2 + 2	1	6	2	>	23 (21)	23 (21)	23 (21)	160–170	47–70
*A. dehuaensis*	343	253	90	21–29%	no	6	2 + 2 (3) + 3 (4)	1	5 (6)	1	>	23	23	23	142–153	63–81
*A.emilyae*	519.5	424.4	95.1	18–20.3%	no	6	2 + 2	1	5	2 (1)	>	23	23	23	157–166	60–65
*A. formosanus*	853	717	136	16%	no	6	2 + 2	0	6–7	2 (1 or 2)	<	27 (29)	25–27	25	158–169	61–83
*A. hainanus*	310	-	80	26–27%	no	6	1 + 2 + 3	1	5	1	≥	23	23	23	165–168	67–69
*A. huangjietangi*	404	340	64	15–23%	no	6	2 + 2 + 3	1	5–6	2	<	23	23	23	157–170	47–67
*A. spinalis*	600	345	83	15–25%	no	6–7	2 + 2 (3)	1	6	1	≤	23 (25)	23 (25)	23 (25)	138–175	39–69
*A. timi*	177.9	140	37.9	21%	no	6	2 + 2	0	6	2 (1)	>	25	25	25	170	72
*A. tranganensis*	448	334	114	25%	no	6	2 + 3	1	6	2	>	25	23	23	171	73+
*A. werner*	550	-	-	25–30%	no	6	2 + ?	1	6	2	=	23 (21)	23 (21)	23 (21)	157–191	67–98
*A. yangdatongi*	397	293	104	26.20%	no	6	2 + 2 + 3	1	6	2	>	23	23	19	161	82
*A. yunkaiensis*	448.1	386.3	63.3	18.5–20.0%	no	6	2 + 2 + 3 (4)	1	6	2	>	23	23	23	151–162	56–79
*A. zugorum*	458	353	105	23%	no	6	2 + 2	0	7	2	>	25	23	23	173	70
*A. jinggangensis*	460	-	80	17–22%	no	6	2 (1) + 2 + 3 (4)	0	6	2 (1)	>	23	23	23	156–164	51–64
*A. juliani*	413	304	109	22–37%	no	6(7)	2 + 2	1	6	2 (2)	>	25	23	23	173–179	77–91
*A. meiguensis*	555	-	81	15–25%	yes	6	2 (3) + 2 (3)	1	6(5)	2 (3)	no suture	21 (23)	19–21 (23)	19	146–173	39–60
*A. niger*	730	-	110	15–18%	no	6	2 + 2	1	6	2	≤	25	25	23	169–185	52–72
*A. ningshanensis*	527	463	72	12–16%	no	6	2 + 2 + 3 (4)	1	5	2	=	23	23	23 (21)	159–174	41–46
*A. panzhihuaensis*	257	194	63	24.60%	Yes	6	2 + 2	1	6	2	no suture	23	23	19	160	73
*A. pingbianensis*	429	345	84	24%	no	7	2 + 2 (+3)	0	7	2 (1)	=	23	23	23	160	56
*A. rufescens*	450	210	80	17–28%	no	6	2 + 2 (3)	1	5	1	>	23	23–25	23	138–165	48–75

Note: Values given in brackets indicate infrequent conditions, TL max. = maximum total length, SVL max. = maximum snout-vent length, TaL max. = maximum tail length, Int. fus.: internasal fused to prefrontal, SPL = supralabials, IFL = infralabials, aTMP [eye contact] = anterior temporals contact eye, LSBI vs. LSBP = length of suture between internasals vs. length of suture between the prefrontals, DSR = dorsal scale rows, V = ventral scales, SC = subcaudals.

## Data Availability

The data presented in this study are available on request from the corresponding author.
